# CmWRKY15-1 Promotes Resistance to Chrysanthemum White Rust by Regulating *CmNPR1* Expression

**DOI:** 10.3389/fpls.2022.865607

**Published:** 2022-04-27

**Authors:** Ge Gao, Ruibing Jin, Di Liu, Xin Zhang, Xiaomei Sun, Pengfang Zhu, Hongyu Mao

**Affiliations:** ^1^College of Forestry, Shenyang Agricultural University, Shenyang, China; ^2^Key Laboratory of Forest Tree Genetics, Breeding and Cultivation of Liaoning Province, Shenyang, China

**Keywords:** chrysanthemum white rust (CWR), resistance, CmWRKY15-1, regulation mechanism, *CmNPR1*

## Abstract

Chrysanthemum white rust (CWR), a disease caused by the fungus *Puccinia horiana* Henn., seriously impairs the production and ornamental value of chrysanthemums. We previously isolated the disease-resistance gene *CmWRKY15-1* from the chrysanthemum and generated *CmWRKY15-1* transgenic plants. Here, we determined that *CmWRKY15-1*-overexpressing lines of the susceptible cultivar ‘Jinba’ show higher defensive enzyme activity and lower H_2_O_2_ levels than a wild type after inoculation with *P. horiana*, indicating that *CmWRKY15-1* positively regulates plant responses to *P. horiana*. To further explore the mechanism underlying this effect, we performed RNA sequencing using the leaves of wild-type and *CmWRKY15-1*-RNA interference lines of the resistant cultivar ‘C029’ after treatment with *P. horiana*. We identified seven differentially expressed genes in the salicylic acid (SA) pathway, including *CmNPR1* (*Non-expressor of pathogenesis-related genes 1*), encoding an important regulator of this pathway. We isolated the *CmNPR1* promoter by hiTAIL-PCR and predicted that it contains pathogen-induced W-box elements. The promoter region of *CmNPR1* was activated by *P. horiana* in a β-glucuronidase activity assay. Yeast one-hybrid assays showed that CmWRKY15-1 binds to the *CmNPR1* promoter region to regulate its expression. Finally, we confirmed the interaction between CmWRKY15-1 and CmNPR1 in a bimolecular fluorescence complementation assay. We propose that CmWRKY15-1 interacts with CmNPR1 to activate the expression of downstream pathogenesis-related genes that enhance resistance to *P. horiana* through the SA pathway. These findings shed light on the mechanism underlying resistance to CWR.

## Introduction

Chrysanthemum (*Chrysanthemum morifolium*) originated in China and is one of 10 flowers traditionally grown there. This plant is an important ornamental flower with considerable economic value globally (Li et al., 2015; Xin et al., 2021). Chrysanthemum white rust (CWR) is an epidemic disease caused by the fungus *Puccinia horiana*. Spores of *P. horiana* spread easily in autumn and winter at temperatures of 4–24°C and quickly infect leaf surfaces during germination (Yoo and Roh, 2014; Keefe and Davis, 2015). This disease has become endemic to most chrysanthemum-growing areas in China, greatly hampering the sale of this crop to the international market (Zeng et al., 2013; Torres et al., 2017). Exploring the molecular mechanisms that result in resistant cultivars could help provide safer and more economical methods for controlling CWR (Alaei et al., 2009).

Plants must respond to numerous environmental stresses and have therefore evolved numerous defense mechanisms to protect themselves throughout their lifecycles (Chen et al., 2020). Plants utilize two strategies to detect pathogens: pathogen-associated molecular pattern (PAMP)-triggered immunity (PTI) and effector-triggered immunity (ETI) (Tsuda and Katagiri, 2010; Resjö et al., 2019). ETI is qualitatively stronger and faster than PTI and often involves a form of localized cell death called the hypersensitive response (HR) (Dodds and Rathjen, 2010). This regional cell necrosis in plants is accompanied by an increased generation of reactive oxygen species (ROS) to help the plant resist pathogen invasion (Yun and Chen, 2011). Following the HR, the plant rapidly produces salicylic acid (SA) and generates a signal in the primary infected tissue that induces the distal end of the uninfected plant to increase its resistance to secondary infection. This process is called systemic acquired resistance (SAR) (Deenamo et al., 2018; Zhang et al., 2018). ROS and SA are very important in preventing pathogen invasion, and ROS are indispensable components of plant responses to stress (Wang Y. J. et al., 2020; Wang Y. Q. et al., 2020). Plants stressed by pathogenic bacteria produce large amounts of ROS, and ROS levels are closely related to improvements in plant resistance. In response to many types of damage, defensive enzymes such as phenylalanine ammonia-lyase (PAL), peroxidase (POD), catalase (CAT), and superoxide dismutase (SOD) also eliminate ROS to reduce oxidative damage (Torun, 2019).

WRKY transcription factors are important regulators of the SA signaling pathway (Zhao N. et al., 2020). *CsWRKY22*, *MdWRKY15*, and *LiWRKY* enhance the resistance of orange (*Citrus sinensis* Osbeck), apple (*Malus* × *domestica*), and Easter lily (*Lilium longiflorum*) to *Xanthomonas citri* subsp. *citri*, *Botryosphaeria dothidea*, and *Botrytis cinerea*, respectively (Zhao X. Y. et al., 2020; Kumari et al., 2021; Long et al., 2021). Heterologous expression of *PtrWRKY73* in Arabidopsis (*Arabidopsis thaliana*) activates the expression of other downstream genes in the SA pathway and enhances resistance to the pathogen *Pseudomonas syringae* pathovar *tomato* strain DC3000 (Pst DC3000) (Duan et al., 2015). We previously identified *CmWRKY15-1*, a gene whose expression is significantly induced upon infection of the disease-resistant chrysanthemum variety ‘C029’ with *P. horiana*, based on transcriptomic data. In addition, *CmWRKY15-1*-overexpressing (OE) lines showed higher SA content and greater resistance to *P. horiana*. This indicates that *CmWRKY15-1* can participate in the regulation of plant disease *via* the SA pathway (Dong et al., 2018; Bi et al., 2021).

WRKY transcription factors regulate gene expression by specifically binding to W-box (TTGACC/T) *cis*-elements in the promoters of their target genes to regulate their transcription and enhance plant defense (Yu et al., 2001; He et al., 2016). NON-EXPRESSOR OF PATHOGENESIS-RELATED GENES 1 (NPR1) is a master regulator involved in SAR. The *NPR1* promoter contains a W-box that binds WRKY transcription factors, suggesting that expression of NPR1 is induced by WRKY or other transcription factors (Li et al., 2018). NPR1 contains two domains associated with protein-protein interactions: the BTB/POZ (broad-complex, tramtrack, and bric-a-brac/pox virus and zinc finger) domain and the ankyrin-repeat (ANK) domain (Chern et al., 2005). Under stable conditions, NPR1 oligomers exist in the cytoplasm *via* intermolecular disulfide bonds. ETI signals facilitate the generation of SA in plants, which results in biphasic redox changes and the phosphorylation and subsequent monomerization of NPR1. This, in turn, allows NPR1 to translocate into the nucleus, where it regulates target gene expression by interacting with WRKY and TGA transcription factors and NIM1-interacting proteins (Mou et al., 2003). *Hordeum vulgare* NPR1 interacts with the HvWRKY6, HvWRKY40, and HvWRKY70 genes to increase resistance to *P. triticina* in barley (Gao et al., 2018).

Although we previously demonstrated that the overexpression of *CmWRKY15-1* enhanced the resistance to pathogens and obtained the transgenic plants of *CmWRKY15-1* (Bi et al., 2021), the mechanism of CmWRKY15-1 and its target gene in the SA pathway is not clear. Therefore, here, we sampled leaves of wild-type (WT) and *CmWRKY15-1*-RNA interference (RNAi) lines of ‘C029’ after inoculation with *P. horiana* and analyzed the samples by transcriptome sequencing (RNA-seq). This analysis identified the differentially expressed gene (DEG) *CmNPR1*. Our results showed that the expression of pathogenesis-related (*PR*) genes significantly decreased in *CmNPR1* silenced lines after inoculation with *P. horiana*. Next, we showed that CmWRKY15-1 binds to the promoter region of *CmNPR1* to regulate its expression. Finally, we determined that CmWRKY15-1 interacts with CmNPR1 to activate the expression of *PR* genes, thereby contributing to resistance to *P. horiana*. These findings shed light on the regulatory mechanisms of *CmWRKY15-1* and *CmNPR1* involved in plant-pathogen interactions *via* the SA signaling pathway. In addition, they provide a valuable reference to help fulfill the long-term goal of breeding disease-resistant chrysanthemum.

## Materials and Methods

### Plant Materials

Using RNAi, we previously generated *CmWRKY15-1*-silenced lines of ‘C029’ and *CmWRKY15-1*-OE lines of ‘Jinba.’ The white rust-resistant chrysanthemum cultivar ‘C029,’ the susceptible cultivar ‘Jinba,’ and various transgenic lines were used as experimental materials. These plant materials were provided by the Forestry College of Shenyang Agricultural University, Shenyang, China, and all experiments were conducted there in 2020 and 2021. Seedlings at the six- to eight-leaf stage were placed in a potting soil mixture and grown in a greenhouse under fluorescent lights for 2 weeks at 25 ± 3^°^C.

### *Puccinia horiana* Inoculation and Sampling

Teliospores of *P. horiana* were collected from the abaxial sides of chrysanthemum leaves infected with white rust and placed in 1 ml sterile water. The concentration of the teliospores was adjusted to 40–60 per field of vision under a BA400 microscope (Motic, Xiamen). Teliospores were cultured according to the protocol by Takatsu (Takatsu et al., 2008). A drop of teliospore suspension was removed with a sterile straw and dropped onto a glass substrate. This was placed on a U-shaped rod in a culture dish covered with wet filter paper. The teliospores were allowed to germinate at 18–21°C for 24 h and resuspended in sterile water with 0.05% (w/v) Tween 20 (pH 4–6.5). The abaxial sides of leaves were sprayed evenly with the solution, and the plants were covered with plastic film and transferred to the dark and 90% relative humidity. After 16–24 h, infected plants were transferred to a growth chamber maintained at 17°C and 50% relative humidity. Leaves were harvested from WT, OE, and RNAi plants at 0 day (before inoculation), and at 24, 48, and 72 h after treatment with *P. horiana*. Leaves were wrapped in aluminum foil, immediately frozen in liquid nitrogen, and stored at –80^°^C for sequencing experiments and to measure physiological indices.

### Diaminobenzidine Staining and H_2_O_2_ Measurements

H_2_O_2_ content in WT and *CmWRKY15-1*-OE lines of ‘Jinba’ leaves was measured at 0, 24, 48, and 72 h after inoculation with *P. horiana*. Leaves were immersed in diaminobenzidine (DAB) staining solution (1 mg/ml 3,3′-DAB, 10 mM Na_2_HPO_4_, and 0.05% Tween 20, pH 3.8) in the dark for 4–8 h and decolorized in 95% (w/v) ethanol. The intensity of brown coloration indicates H_2_O_2_ content. Quantitative H_2_O_2_ measurements were performed using an Amplex Red hydrogen peroxide/peroxidase assay kit (Solarbio Science and Technology, Beijing). Absorbance was measured using a Tecan infinite F200/M200 plate reader at 560 nm. H_2_O_2_ concentration is indicated in μmol/g fresh weight. The experiment was performed with at least three independent replicates.

### Physiological Indices

Frozen leaf samples of WT and *CmWRKY15-1*-OE lines of ‘Jinba’ were used to measure the activity of defense enzymes, namely, PAL, POD, CAT, and SOD. SOD and POD activities were measured following the protocol by Sun et al. (2013). CAT and PAL activities were measured following the protocol by Kwon and Liu (Kwon and Nguyen, 2003; Liu et al., 2005). All experiments were performed with three biological replicates.

### RNA Extraction, cDNA Library Construction, and Sequencing

The Illumina HiSeq-2000 platform (Illumina Inc., San Diego, CA, United States) by Wuhan MetWare Biotechnology Co., Ltd., (^1^ Wuhan, China) was used for RNA-seq. A total of 12 high-quality RNA samples [six from before inoculation (0 h) and six from after inoculation] were prepared from the leaves of WT and *CmWRKY15-1*-RNAi lines of ‘C029.’ First, samples were obtained from uninoculated WT and *CmWRKY15-1*-RNAi lines of ‘C029’ (0 h). Three samples were collected per strain for a total of six samples (labeled W0 and R0). WT and *CmWRKY15-1*-RNAi lines were then sampled at 24, 48, and 72 h after inoculation with *P. horiana*. Samples at specific time points were combined to form six inoculated samples, labeled WJ and RJ. Because most eukaryotic mRNAs have poly-A tails, mRNAs were enriched using oligo(dT) magnetic beads. Examination of RNA quality, cDNA library construction, and Illumina sequencing library construction were performed as previously described (Ana et al., 2016; Chuang et al., 2020).

### Identification and Functional Analysis of Differentially Expressed Genes

We assembled pathogen-sequence-free reads from the above RNA-seq data into contigs using the Trinity program. All contigs were analyzed with RNA-seq by Expectation-Maximization software to map the clean reads of each sample to the transcriptional reference sequence obtained *via* Trinity splicing (Michael et al., 2014). Next, we obtained the raw counts for each unigene and used the DESeq R package to identify DEGs between W0 vs. WJ, R0 vs. RJ, and WJ vs. RJ. The parameter standards included a cut-off of |log_2_(fold change)| > 1 and a false discovery rate < 0.05. Finally, we analyzed unigene sequences from the NCBI non-redundant (NR) database and euKaryotic Ortholog Groups (KOG) databases and annotated DEGs between W0 vs. WJ, R0 vs. RJ, and WJ vs. RJ using the Gene Ontology (GO) and Kyoto Encyclopedia of Genes and Genomes (KEGG) pathway databases.

### Quantitative Reverse Transcription Polymerase Chain Reaction

To confirm the RNA-seq results, seven disease-resistance DEGs in the SA pathway were selected for verification by quantitative reverse transcription PCR (RT-qPCR). Primers used are shown in Supplementary Table 4. Leaves of the ‘C029’ WT and *CmWRKY15-1*-RNAi lines were harvested at 0 and 48 h after treatment with *P. horiana*. A PrimeScript™ RT reagent Kit with gDNA Eraser (Perfect Real Time), PrimeScript™ II 1st Strand cDNA Synthesis Kit, and SYBR^®^ Premix ExTaq™ II were purchased from Takara. RT-qPCR was performed using an SYBR^®^ Premix ExTaq™ II, following the protocols of the manufacturer. The 2^–ΔΔ^*^CT^* method was used to calculate the expression levels of these disease resistance DEGs (Livak and Schmittgen, 2008). All experiments were performed with three biological replicates.

### Isolation and Sequencing Analysis of *CmNPR1*

One DEG, *CmNPR1*, was identified by sequencing. The coding sequence (CDS) of *CmNPR1* was cloned from ‘C029’ by PCR with *CmNPR1* forward (F) and reverse (R) primers and was cloned into pEASY^®^-T1 using a Simple Cloning Kit (TransGen Biotech, Beijing). Primers are shown in Supplementary Table 4. This vector was transformed into *Escherichia coli* DH5α (Aidlab) and clones harboring the inserts were identified for subsequent experiments. The cDNA and deduced protein sequences from *CmNPR1* were analyzed using DNAMAN software. The online tool ExPASy^2^ was used to predict the physicochemical properties of *CmNPR1*, and the SOPMA^3^ website and Phyre^2^ database were used to predict protein structure.^4^ Finally, MEGA 5.0 software was used for the phylogenetic analysis of CmNPR1 proteins.

### Functional Analysis of *CmNPR1*

To investigate the potential function of *CmNPR1*, the silencing vector pTRV2-*CmNPR1* was constructed, and expression of the gene was silenced using the virus-induced gene silencing (VIGS) technique in ‘C029.’ Leaves of WT and TRV-*NPR1* lines were sampled at 0, 24, 48, and 72 h after inoculation with *P. horiana*. The relative expression levels of *CmPR1*, *CmPR2*, and *CmPR10* were quantified by RT-qPCR. All experiments were performed with three biological replicates. Primers are shown in Supplementary Table 4.

### Isolation of the *CmNPR1* Promoter and Glucuronidase Staining

The primers NPR1SP1, NPR1SP2, and NPR1SP3 were synthesized by hiTAIL-PCR following the protocol of the manufacturer (Chen and Liu, 2007). Through genome walking, we obtained the 1,685-bp promoter region of *CmNPR1* upstream of the ATG codon. Promoter elements of *CmNPR1* were analyzed using the Plant CARE^5^ and PLACE^6^ websites. The *CmNPR1* promoter was amplified by PCR with specific primers CmNPR1.1-F/R, CmNPR1.2-F/R, and CmNPR1.3-F/R (based on the predicted *cis*-acting elements in this promoter) using ‘C029’ DNA as a template. The 1,685-bp *CmNPR1* promoter region was obtained, along with truncated sequences 850 and 450 bp long. Primers used are shown in Supplementary Table 4. These sequences were ligated into the pCAMBIA1301 vector, replacing the 35S promoter, to form the recombinant expression vectors *pNPR1.1:GUS*, *pNPR1.2:GUS*, and *pNPR1.3:GUS*, respectively. The vectors were introduced into *Agrobacterium tumefaciens* strain EHA105. Combination vectors were transformed into ‘C029’ *via Agrobacterium*-mediated transient transformation. Leaves of WT and transgenic plants at 0, 24, 48, and 72 h after treatment with *P. horiana* were subjected to β-glucuronidase (GUS) staining using a GUS staining kit (Real-Times Biotechnology Co., Ltd., Beijing, China). All experiments were performed with three biological replicates.

### Yeast One-Hybrid Assay

Yeast one-hybrid (Y1H) assays were conducted using the Matchmaker™Gold Yeast One-Hybrid System (Clontech, Palo Alto, CA, United States) for experimental validation. The full-length 1,685-bp *CmNPR1* promoter was ligated into the pAbAi vector, generating p*NPR1*-AbAi. The linearized vector was digested with a single endonuclease and transfected into yeast strain Y1HGold and used as bait. The CDS of CmWRKY15-1 was cloned into pGADT7 to generate the prey vector pGAD-CmWRKY15-1; the pGADT7 vector and bait constructs were transformed into yeast cells as a negative control. Subsequently, all constructs were transformed into the Y1HGold strain. Finally, the colonies were plated on synthetic defined (SD) medium without leucine (SD/–Leu) and SD/–Leu supplemented with 800 ng/ml aureobasidin A (AbA) and allowed to grow for 3 days at 28°C. The *CmNPR1* promoter was divided into two segments based on analysis of the *cis*-acting elements PA1 and PA2. PA1 contains the critical regulatory W-box element of the *CmNPR1* promoter. Two fragments were cloned into the pAbAi vector to produce new recombinant vectors pA1-AbAi and pA2-AbAi. These two bait vectors, and the prey vector pGAD-CmWRKY15-1, were transferred into Y1HGold. The transformants were grown on medium with 800 ng/ml SD/–Leu with AbA and cultivated at 28–30°C for 3 days. All experiments were performed with three biological replicates. Primers are shown in Supplementary Table 4.

### Bimolecular Fluorescence Complementation Assay

Bimolecular fluorescence complementation (BiFC) vectors were constructed for the transient transformation of *Nicotiana benthamiana*. The CDSs of CmNPR1 and CmWRKY15-1 were cloned into the p2YC and p2YN vectors using the BiFC-NPR1-F1/BiFC-NPR1-R1 and BiFC-WRKY15-1-F1/BiFC-WRKY15-1-R1 primers *via Pac*I/*Spe*I, generating p2YC-CmNPR1 and p2YN-CmWRKY15-1, respectively. Cells of *A. tumefaciens* strain GV3101 carrying the BiFC constructs were infiltrated into 4- to 5-week-old *N. benthamiana* leaves. Yellow fluorescent protein (YFP) signals in the *N. benthamiana* leaf cells were observed under a laser scanning confocal microscope. All experiments were performed with three biological replicates. Primers are shown in Supplementary Table 4.

### Statistical Analysis

Data were analyzed using ANOVA and *t*-tests to determine significant differences with SPSS 24.0 software.

## Results

### *CmWRKY15-1* Functions in Resistance to *Puccinia horiana* by Regulating Reactive Oxygen Species Levels

**FIGURE 1:**
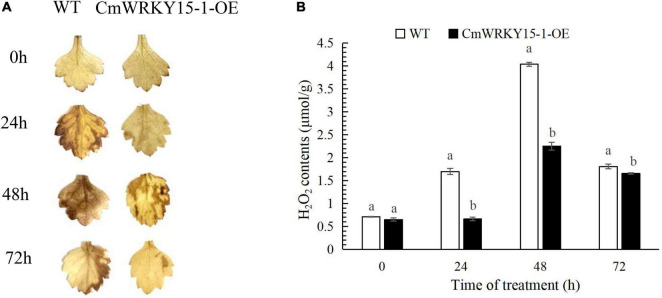
Changes in the levels of H_2_O_2_ in wild-type (WT) and *CmWRKY15-1-*overexpressing (OE) lines of ‘Jinba.’ **(A)** DAB staining of detached leaves to observe H_2_O_2_ levels. **(B)** Measurement of H_2_O_2_ content in leaves. Different letters demonstrate significantly different by Duncan’s multiple range test at *p* < 0.05.

To determine whether ROS are involved in resistance to *P. horiana*, we performed DAB staining to measure the H_2_O_2_ content in leaves. More H_2_O_2_ accumulated in WT than in *CmWRKY15-*1-OE lines after inoculation with *P. horiana* (Figure 1A). We also quantified endogenous H_2_O_2_ content. The levels of H_2_O_2_ increased over time and peaked at 48 h in WT and *CmWRKY15-1*-OE lines after inoculation with *P. horiana*. Compared with the WT, *CmWRKY15-1*-OE lines had lower H_2_O_2_ levels after inoculation with *P. horiana*, which is consistent with the results of DAB staining (Figure 1B). This result indicated that OE *CmWRKY15-1* enhanced plants’ resistance to *P. horiana* by inhibiting the accumulation of ROS.

### Overexpression of *CmWRKY15-1* Resulted in Increased Enzyme Activities

**FIGURE 2:**
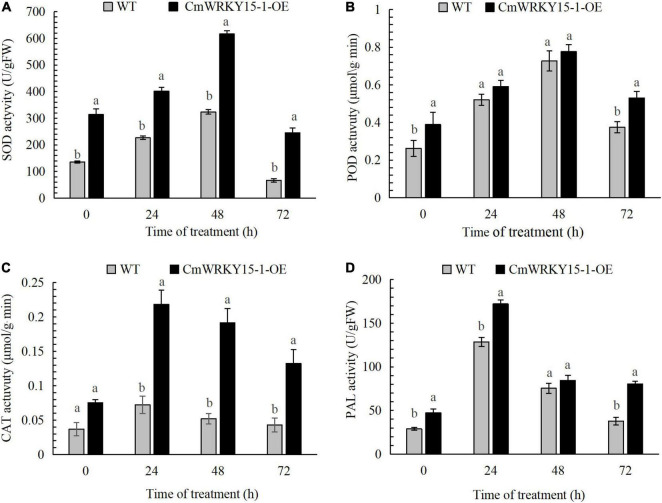
Activity of defense enzymes in WT and *CmWRKY15-*1*-*OE lines of ‘Jinba.’ **(A)** SOD activity. **(B)** POD activity. **(C)** CAT activity. **(D)** PAL activity. Error bars show SD of three replicates. Different letters demonstrate significantly different by Duncan’s multiple range test at *p* < 0.05.

We measured PAL, POD, SOD, and CAT activities in leaf samples leaves from WT and *CmWRKY15-1*-OE lines of ‘Jinba.’ The activities of all four enzymes showed an upward trend and then a decrease at 72 h both in WT and *CmWRKY15-1*-OE lines following inoculation with *P. horiana*. The activities of these enzymes were significantly higher in *CmWRKY15-1*-OE lines than in the WT. SOD and POD activities reached their highest levels at 48 h in both WT and *CmWRKY15-1*-OE lines. CAT and PAL activities peaked at 24 h in WT and *CmWRKY15-1*-OE lines (Figure 2). Our results showed that the overexpression of *CmWRKY15-1* helps to activate defense systems to respond to infection with *P. horiana*.

### RNA-Seq Data Analysis

Approximately 100.8 GB of clean reads were acquired from 12 samples. The average Q20 and Q30 values of the clean reads were 95.56% and 89.94%, respectively, showing that all clean reads were of high quality (Supplementary Table 1). We successfully annotated 272,669 unigenes *via* comparisons with databases, namely, KOG, NR, Swiss-Prot, GO, KEGG, Pfam, and TrEMBL. Results are shown in Supplementary Table 2. We performed a statistical analysis of the unigenes with homologs in the NR database to determine the species distribution of homologous sequences. The top three species with the most annotated homologous genes were sunflower (*Helianthus annuus*), lettuce (*Lactuca sativa*), and cardoon (*Cynara cardunculus*), accounting for 35.56, 19.09, and 16.63% of the total, respectively (Supplementary Figure 1). We also annotated the 53,463 unigenes in the KOG database. The highest number of genes (11,233) belonged to the R category: General Function Prediction Only (Supplementary Figure 2).

### Differentially Expressed Genes Were Analyzed Among Three Comparison Pairs

To achieve a comprehensive insight into the functions and mechanisms of DEGs in ‘C029’ after inoculation with *P. horiana*, we analyzed the DEGs among three comparison pairs: W0 vs. WJ, R0 vs. RJ, and WJ vs. RJ. The expression levels of 5,449 and 6,128 genes significantly differed in W0 vs. WJ and in R0 vs. RJ, respectively. In addition, we also found 1,684 DEGs in WJ vs. RJ. To explore the functions of these DEGs, they were annotated using GOseq. The annotations were grouped into three categories: cellular components, molecular functions, and biological processes. In the cellular components category, the “cell” and “cell part” subclasses were the most enriched in W0 vs. WJ, R0 vs. RJ, and WJ vs. RJ. In the molecular functions category, the most highly enriched GO terms were “catalytic activity” and “binding” in W0 vs. WJ, R0 vs. RJ, and WJ vs. RJ (Supplementary Figure 3). Analysis of the biological process category showed that the “cellular process” and “metabolic process” subclasses were the most abundant in W0 vs. WJ, R0 vs. RJ, and WJ vs. RJ.

We used KEGG enrichment pathway analysis to explore the molecular mechanisms of these DEGs. Most of the DEGs in W0 vs. WJ, R0 vs. RJ, and WJ vs. RJ belonged to the KEGG pathways “metabolic pathway” and “biosynthesis of secondary metabolites” (Supplementary Figure 5). A Venn diagram created to visualize the number of DEGs between W0 vs. WJ, R0 vs. RJ, and WJ vs. RJ revealed an overlap of 65 DEGs (Supplementary Figure 4).

### Changes in Candidate Differentially Expressed Genes Expression Are Closely Related to the RNA-Seq Data

**FIGURE 3:**
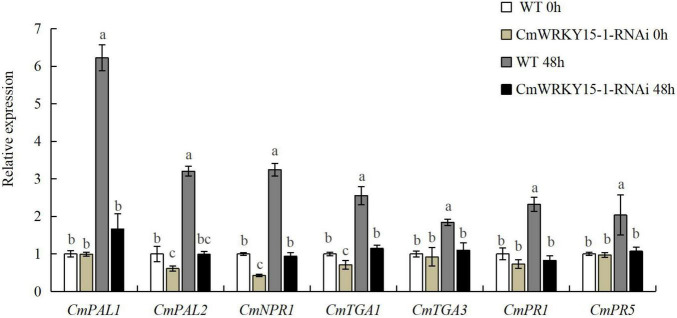
Expression of differentially expressed genes (DEGs) in wild-type (WT) and *CmWRKY15-1*-RNA interference (RNAi) lines of ‘C029.’ Error bars show standard deviation of three replicates. Different letters demonstrate significantly different by Duncan’s multiple range test at *p* < 0.05.

To validate the reliability of DEGs identified by RNA-seq analysis, we analyzed the expression levels of seven candidate genes in the SA signaling pathway from the 65 DEGs between W0 vs. WJ, R0 vs. RJ, and WJ vs. RJ *via* RT-qPCR (Supplementary Table 3). These genes included two SA synthetic signaling pathway genes (*CmPAL1* and *CmPAL2*), three SA-response-related genes (*CmPR1*, *CmPR5*, and *CmNPR1*), and two TGA transcription factor genes (*CmTGA1* and *CmTGA3*). Our results showed that the RT-qPCR data were closely related to the RNA-seq data. In the silenced lines, expression of DEGs was inhibited, as their expression levels were always lower in these lines than in the WT (Figure 3). One DEG, *CmNPR1*, is an important regulator and a core point in the downstream signal transduction network of plant disease resistance. Expression of this gene significantly increased in both the WT and *CmWRKY15-1*-RNAi lines after treatment with *P. horiana*.

### Isolation and Sequence Analysis of *CmNPR1*

**FIGURE 4:**
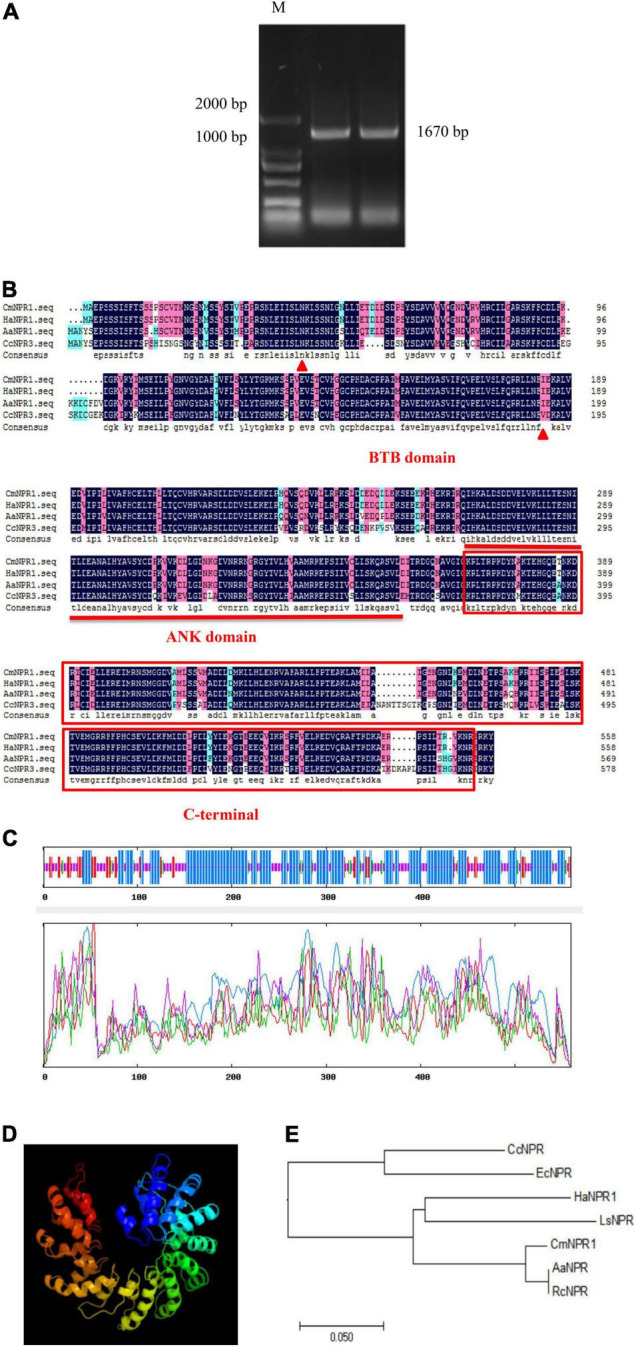
**(A)** PCR amplification product of *CmNPR1*: M, Marker. **(B)** Sequence alignment analysis between CmNPR1 and other several related NPR1 proteins from other Asteraceae. Triangle, N-terminal BTB/POZ domain; straight line, ANK domain; box, C-terminal NPR1/NIM1 region. Cm, *Chrysanthemum morifolium*; Ha, *Helianthus annuus* L.; Aa, *Artemisia annua*; Cc, *Cynara cardunculus* L. **(C)** The predicted secondary structure of CmNPR1. Blue, alpha helix; red, folding and extending chain; green, beta turn; yellow, random coil. **(D)** The predicted three-dimensional structure of CmNPR1. **(E)** Phylogenetic analysis of CmNPR1. Rc, *Ricinus communis*; Ls, *Lactuca sativa*; Ec, *Erigeron canadensis*.

We cloned the CDS of *CmNPR1* from ‘C029,’ showing that it is 1,670 bp long and encodes a 558-amino-acid protein (Figure 4A). Analysis of the *CmNPR1* protein sequence revealed that it has 97, 77, and 78% sequence similarity to the NPR protein sequences from sweet wormwood (*Artemisia annua*), *H. annuus*, and *C. cardunculus*, respectively (Figure 4B), confirming that this gene is *CmNPR1*. According to the predicted results, the *CmNPR1* protein N-terminal region includes an evident BTB/POZ domain between amino acids 137 and 552; this domain is a potential target of the Cullin3-E3 ligase ubiquitin degradation pathway. An ANK domain was present in the middle region of this protein at amino acids 802–1,059. This domain is responsible for mediating the interaction between NPR1 and WRKY or TGA. The C-terminal NPR1/NIM1 region (between amino acids 1,102 and 1,632) and the N-terminal BTB/POZ domain participate in the binding of NPR1 to components of the SA signaling pathway.

Analysis using ExPASy revealed that NPR1 protein primarily comprises leucine, serine, valine, glutamic acid, aspartic acid, and lysine residues, which account for 12.2, 9.1, 8.2, 7.5, 7.2, and 7.0% of the amino acid residues, respectively. The molecular formula of the protein is C_2774_H_4501_N_773_O_847_S_29_. Based on an analysis of the predicted protein structure, we submitted the CmNPR1 protein sequences to SOPMA to predict its secondary structure. Of the 310 alpha-helices present in CmNPR1, 176 were part of a random coil, 52 were part of an extension strand, and 21 were part of a beta turn, accounting for 55.46, 31.48, 9.30, and 3.76% of the protein, respectively (Figure 4C). We predicted its tertiary structure using the Phyre^2^ database (Figure 4D). In addition, a comparison of CmNPR1 with other proteins from various species indicated that it is most similar to AaNPR from *A. annua* and RcNPR1 from the castor bean (*Ricinus communis*) (Figure 4E).

### *CmNPR1* Silencing Resulted in Decreased Expression of Pathogenesis-Related Genes

**FIGURE 5:**
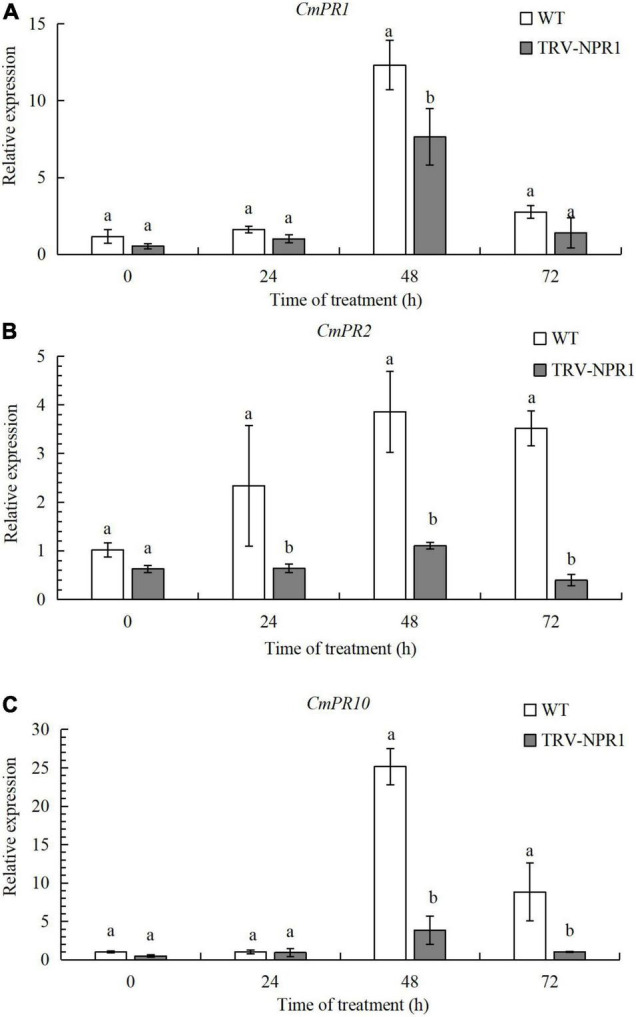
Transcript levels of *CmPR1* **(A)**, *CmPR2* **(B)**, and *CmPR10* **(C)** genes determined by RT-qPCR. Error bars show SD of three replicates. Different letters demonstrate significantly different by Duncan’s multiple range test at *p* < 0.05.

To verify the function of *CmNPR1*, we obtained *CmNPR1*-silenced lines (TRV-NPR1) of ‘C029’ generated by VIGS. We then performed RT-qPCR to identify any changes in the expression of these downstream *PR* genes associated with plant defense in WT and TRV-NPR1 lines. Three genes, *CmPR1*, *CmPR2*, and *CmPR10*, showed lower expression in the silenced plants than in the WT controls at 0, 24, 48, and 72 h. Transcript levels of all three genes initially increased, peaked at 48 h, and then decreased in both WT and TRV-NPR1 lines (Figure 5). The results showed that *CmNPR1* can respond to infection with *P. horiana* and induce the expression of *PR* genes.

### The *CmNPR1* Promoter Can Respond to *Puccinia horiana* Infection

**FIGURE 6:**
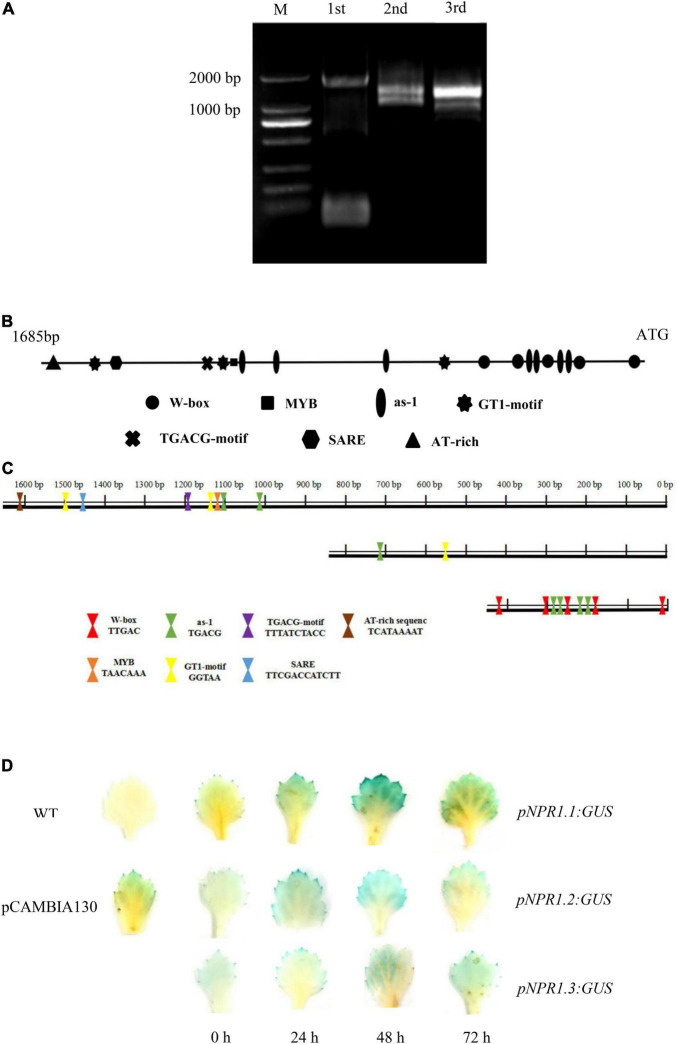
Analysis of the *CmNPR1* promoter. **(A)** Amplification products of the *CmNPR1* promoter obtained by hiTAIL-PCR: M, Marker; 1st: First round PCR; 2nd: Second round PCR; 3rd: Third round PCR. **(B)** Analysis of *cis*-elements in the *CmNPR1* promoter. **(C)** Diagram of the pNPR1.1, pNPR1.2, and pNPR1.3 vectors. **(D)** GUS histochemical staining to analyze expression of the *CmNPR1* promoter in response to the pathogen. WT, wild-type negative control; pCAMBIA1301, positive control.

We cloned the 1,685-bp promoter of *CmNPR1* by hiTAIL-PCR (Figure 6A). We analyzed *cis*-acting elements in the *CmNPR1* promoter using the Plant CARE and PLACE databases. Numerous motifs were discovered in the CmNPR1 promoter, including core *cis*-acting elements: 13 transcription start site TATA-boxes; 19 CAAT-boxes; one MYB element (GA-responsive); one SARE sequence; seven as-1 (SA-responsive) elements; one TGACG motif [methyljasmonate (MeJA)-responsive]; one AT-rich sequence (stress-responsive); three GT1 motifs (light responsive); and five W-boxes (Figure 6B). The W-box is a binding site for WRKY transcription factors, suggesting that the promoter of *CmNPR1* may be regulated by the transcription factor CmWRKY15-1. Based on analysis of *cis*-acting elements, using ‘C029’ DNA as a template, we constructed three expression vectors with the *GUS* gene driven by three promoter sequences and obtained by PCR (in place of the *Cauliflower mosaic virus* 35S promoter): the full-length *CmNPR1* promoter sequence and two truncated sequences (Figure 6C). To explore the mechanism of *CmNPR1* promoter fragments on *P. horiana* response, we transiently transformed three promoter fragments into ‘C029.’ We sampled leaves at different time points after inoculation with *P. horiana* and evaluated GUS activity. Little GUS staining was observed in any of the transgenic leaves at 0 h. Compared with the mock control, the three transgenic leaves (harboring *pNPR1.1:GUS*, *pNPR1.2:GUS*, and *pNPR1.3:GUS*) gradually turned blue with increasing time after inoculation, with the deepest staining observed at 48 h, after which staining decreased again. GUS activity driven by the *CmNPR1* promoter was significantly upregulated after treatment with *P. horiana*, and GUS activity driven by the *CmNPR1.1* promoter (*pNPR1.1: GUS*) was significantly higher than that of *pNPR1.2:GUS* and *pNPR1.3:GUS* (Figure 6D). We propose that *CmNPR1* has a pathogen-inducible promoter and can be induced by *P. horiana*. In addition, the change in *CmNPR1* expression levels in ‘C029’ leaves after treatment with *P. horiana* is closely related to its change in promoter activity.

### *CmNPR1* Expression Can Be Regulated by CmWRKY15-1

**FIGURE 7:**
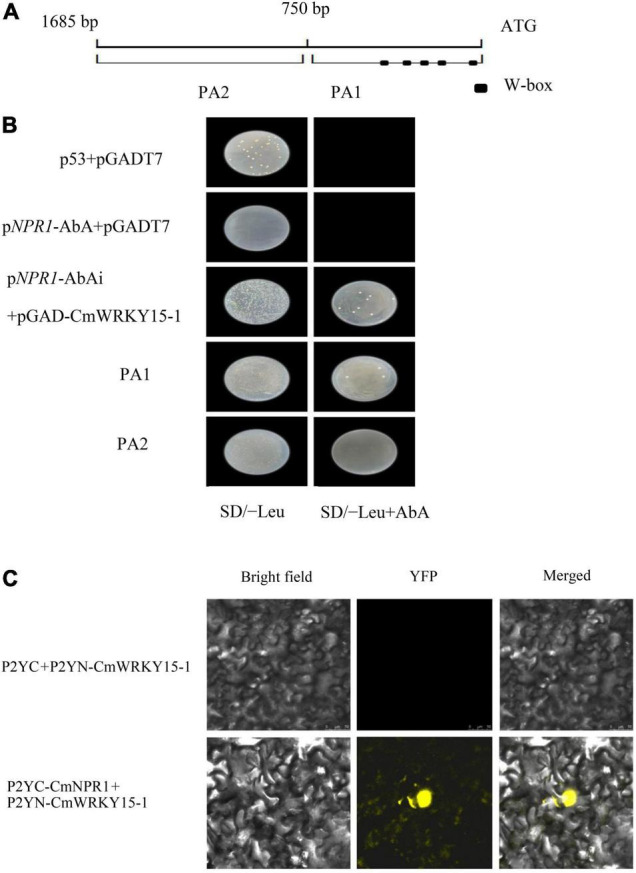
Regulatory relationship between *CmWRKY15-1* and *CmNPR1*. **(A)** Schematic diagram of the 1,600-bp *CmNPR1* promoter. PA1, 0 to –750 bp; PA2, –750 to –1,685 bp; squares indicate W-box elements. **(B)** Yeast one-hybrid (Y1H) analysis of CmWRKY15-1 binding to the *CmNPR1* promoter. **(C)** Fluorescence confocal detection of *Nicotiana benthamiana* mesophyll cells. Bright field, visible images; YFP, yellow fluorescent protein images; Merged, merged fluorescence, and visible images.

The interaction between the promoter of full-length *CmNPR1* and the CmWRKY15-1 protein was verified with a Y1H assay. We transferred the bait (p*NPR1*-AbAi.) and prey (pGAD-CmWRKY15-1) vectors into Y1HGold yeast cells; pGADT7 and p53 were used as a positive control. The strain was able to grow on plates of SD/-Leu medium supplemented with 800 ng/ml AbA when p*NPR1*-AbAi was coexpressed with pGAD-CmWRKY15-1 in yeast (Figure 7B). The *CmNPR1* promoter was divided into two fragments, PA1 and PA2, and the promoter region PA1 contains W-box elements (Figure 7A). We transformed Y1HGold cells with the pPA1-AbAi or pPA2-AbAi bait vector and the pGAD-CmWRKY15-1 prey vector and plated the cells on SD/-Leu medium containing 800 ng/ml AbA. Y1H assays showed that CmWRKY15-1 interacted with fragment PA1 but not PA2 (Figure 7A). Therefore, CmWRKY15-1 can bind to the W-box elements in the promoter of its target gene *CmNPR1*. These results show that CmWRKY15-1 has DNA binding activity and specifically binds to the *CmNPR1* promoter.

### CmWRKY15-1 Can Interact With CmNPR1

Finally, to examine the interaction between CmWRKY15-1 and CmNPR1, we performed a BiFC analysis. We cloned the CDS of *CmWRKY15-1* and CmNPR1 into vectors containing the N-terminal of p2YN and C-terminal regions of p2YC and then cotransfected *N. benthamiana* leaves with these constructs (p2YC-CmNPR1 and p2YN-CmWRKY15-1). The p2YC vector coexpressed with p2YN-CmWRKY15-1 vectors served as the negative controls. YFP signal was observed only in *N. benthamiana* leaves coexpressing p2YC-CmNPR1 and p2YN-CmWRKY15-1, but not in cells coexpressing the negative control constructs (Figure 7C). This indicates that CmWRKY15-1 and CmNPR1 interact *in vivo*.

## Discussion

Chrysanthemum is an important ornamental flower in China and around the world. ‘C029’ is a chrysanthemum cultivar with broad-spectrum resistance to pathogenic bacteria and fungi, including CWR due to the fungus *P. horiana*, which causes serious losses in chrysanthemum. *Via* RNA-seq analysis, we had previously isolated the disease resistance gene *CmWRKY15-1* from chrysanthemum following *P. horiana* infection and determined that SA content increased significantly after pathogen infection in *CmWRKY15-1*-OE lines. In addition, *CmWRKY15-1*-OE lines showed higher resistance to *P. horiana*. These results show that *CmWRKY15-1* positively regulates resistance to *P. horiana* by regulating the levels of SA.

Plant defense responses involve complex signal transduction mechanisms. The activation of SAR is accompanied by the production of several signaling molecules. Pathogens induce SA biosynthesis and downstream defense responses by activating *R*-gene-mediated defense signaling (Chen et al., 1993; Gonzalez et al., 2015). In addition, activation of the *R* gene induces ROS production and HR at the site of pathogen infection (Ji et al., 2021). In this study, we observed that more ROS accumulated in WT than in *CmWRKY15-1*-OE lines of ‘Jinba’ after *P. horiana* inoculation. We also quantified endogenous H_2_O_2_ content and found that, as expected, it was higher in WT than in *CmWRKY15-1*-OE lines of ‘Jinba’ after *P. horiana* inoculation, which is consistent with the above findings. Our results suggest that *CmWRKY15-1* participates in plant resistance to *P. horiana* by regulating ROS levels.

The activities of defensive enzymes such as SOD, CAT, POD, and PAL are generally an indicator of plant resistance to stress (Choudhury et al., 2017). Given that *CmWRKY15-1*-OE lines of ‘Jinba’ accumulated less H_2_O_2_, we measured the activities of these four defense enzymes in WT and *CmWRKY15-1*-OE lines after pathogen infection. The activities of all four enzymes were higher in *CmWRKY15-1*-OE lines than in WT after inoculation with *P. horiana*. In particular, PAL activity increased significantly after the inoculation and was significantly higher in *CmWRKY15-1*-OE lines than in WT. In addition, we showed that the *CmPAL1* and *CmPAL2* expression was upregulated after treatment with *P. horiana*. This suggests that SA may be synthesized through the PAL pathway to help plants resist *P. horiana* infection.

To further explore the resistance mechanism, we performed RNA-seq and identified one DEG, *CmNPR1*, between WT lines of the *P. horiana*-resistant cultivar ‘C029’ and ‘C029’ lines with *CmWRKY15-1* silenced by RNAi. NPR1 is a key regulator of the SAR signal transduction pathway downstream of SA (Ding et al., 2018). Change in NPR1 expression affects the generation of SAR in plants; in the absence of NPR1, plants cannot respond to various SAR inducers, and *PR* genes are rarely expressed. In addition, NPR1 induces various defensive reactions, causing plants to exhibit a broad spectrum of resistance responses (Fu and Dong, 2013; Pajerowska-Mukhtar et al., 2013). *AtNPR1* and its orthologs in various plants enhance resistance to biotrophic fungal and bacterial pathogens (Le et al., 2009; Nic-Matos et al., 2017). NPR1 improves disease resistance in *Arabidopsis*, rye (*Secale cereale*), and Cape periwinkle (*Catharanthus roseus*) by regulating the expression of *PR* genes (Hui et al., 1994; Yu et al., 2017; Sung et al., 2019). In this study, when we cloned the *CmNPR1* gene from chrysanthemum ‘C029’ and obtained *CmNPR1*-silenced ‘C029’ lines, they showed decreased levels of *PR* gene expression after infection with *P. horiana*. These results indicate that *CmNPR1* participates in the response of chrysanthemum to white rust by regulating the expression of *PR* genes. Furthermore, we isolated the *CmNPR1* promoter and demonstrated that it responds to *P. horiana*, as revealed by GUS staining. We also showed that CmWRKY15-1 can bind to the *CmNPR1* promoter region to activate its expression. Finally, the interaction between CmNPR1 and CmWRKY15-1 was verified by BiFC.

**FIGURE 8:**
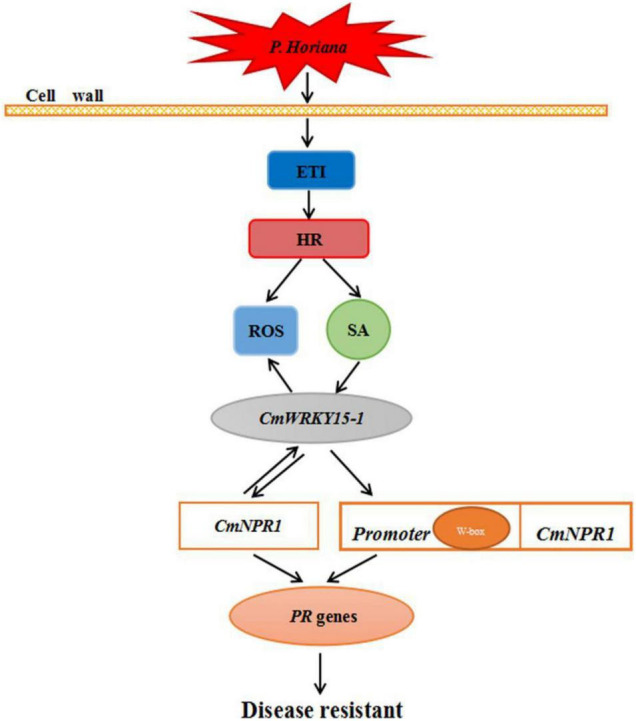
Hypothetical model of the mechanism of ‘C029’ resistance to *Puccinia horiana.*

We propose a model for the mechanism underlying the defense response against *P. horiana* in ‘C029.’ When the plant is infected by *P. horiana*, ETI is triggered to modulate the level of ROS to resist *P. horiana* invasion. At the same time, SA is synthesized *via* the PAL pathway and promotes CmNPR1 interaction with CmWRKY15-1, thereby regulating a change in the expression of downstream *PR* genes to improve disease resistance. In addition, CmWRKY15-1 might also bind to the W-box in the *CmNPR1* promoter to activate its expression. This could result in a change in *PR* gene expression, thereby enhancing resistance to *P. horiana* (Figure 8). Our future research will focus on screening and identification of upstream genes of the SA pathway. Our results convey new insights into the mechanism of plant defense responses and provide evidence for the suitability of ‘C029’ as an important resource for chrysanthemum breeding.

## Data Availability Statement

The datasets presented in this study can be found in online repositories. The names of the repository/repositories and accession number(s) can be found below: National Center for Biotechnology Information (NCBI) BioProject database under accession number PRJNA797165.

## Author Contributions

HM and GG designed the study and wrote the manuscript. GG and RJ performed most of the experiments. DL, XZ, XS, and PZ analyzed the data and discussed the article. All authors revised and approved the manuscript.

## Conflict of Interest

The authors declare that the research was conducted in the absence of any commercial or financial relationships that could be construed as a potential conflict of interest.

## Publisher’s Note

All claims expressed in this article are solely those of the authors and do not necessarily represent those of their affiliated organizations, or those of the publisher, the editors and the reviewers. Any product that may be evaluated in this article, or claim that may be made by its manufacturer, is not guaranteed or endorsed by the publisher.

## Footnotes

1
www.metware.cn

2
https://prosite.expasy.org/

3
https://npsa-prabi.ibcp.fr/

4
http://www.sbg.bio.ic.ac.uk/phyre2/html/

5
http://bioinformatics.psb.ugent.be/webtools/plantcare/html/

6
https://www.dna.affrc.go.jp/PLACE/
