# Supplementary material for: CmWRKY15-1 Promotes Resistance to Chrysanthemum White Rust by Regulating CmNPR1 Expression
Source: Front Plant Sci. 2022 Apr 27;13:865607. doi: 10.3389/fpls.2022.865607 (PMC9094113; doi:10.3389/fpls.2022.865607)
Supplement: Supplementary file 1 [file Data_Sheet_1.docx]

Supplementary Material


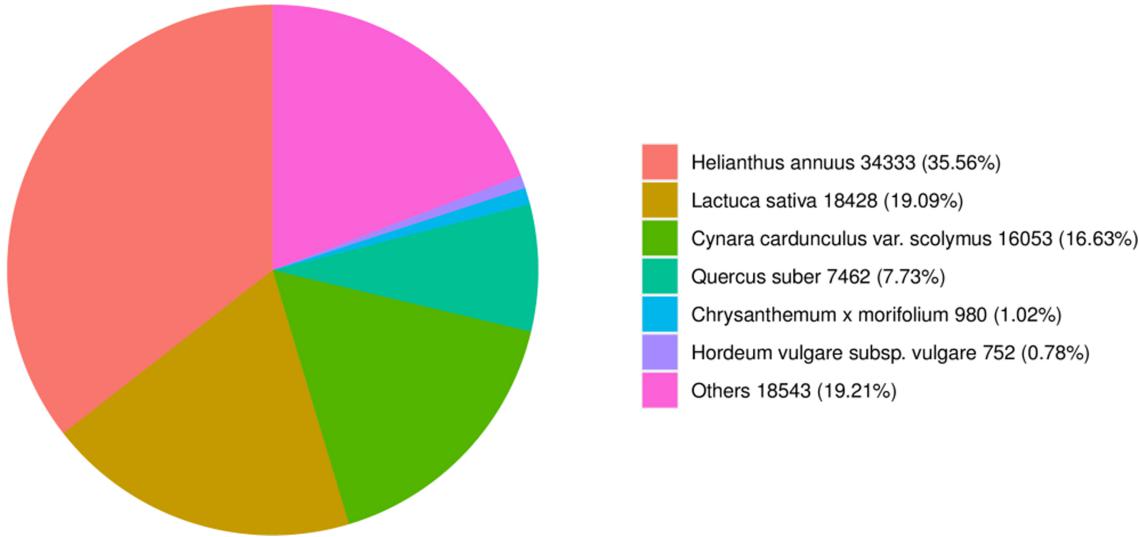


**Supplementary Figure 1.** Species distribution of unigenes in the NR database.


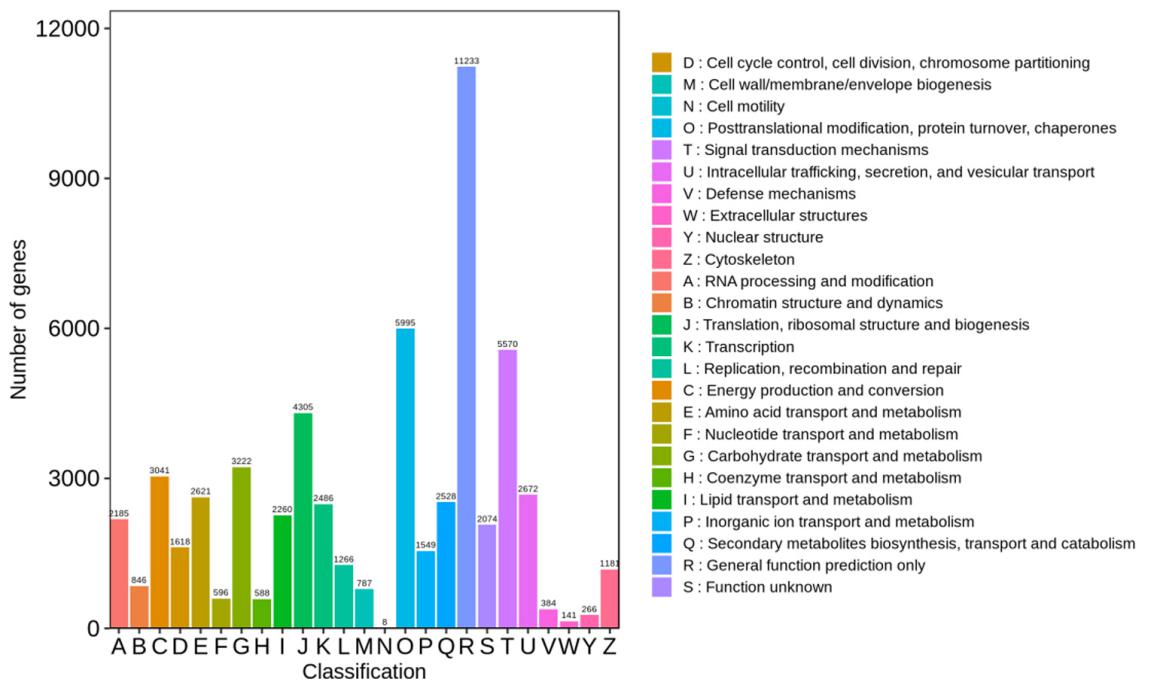


**Supplementary Figure 2.** KOG functional classification of unigene sequences.


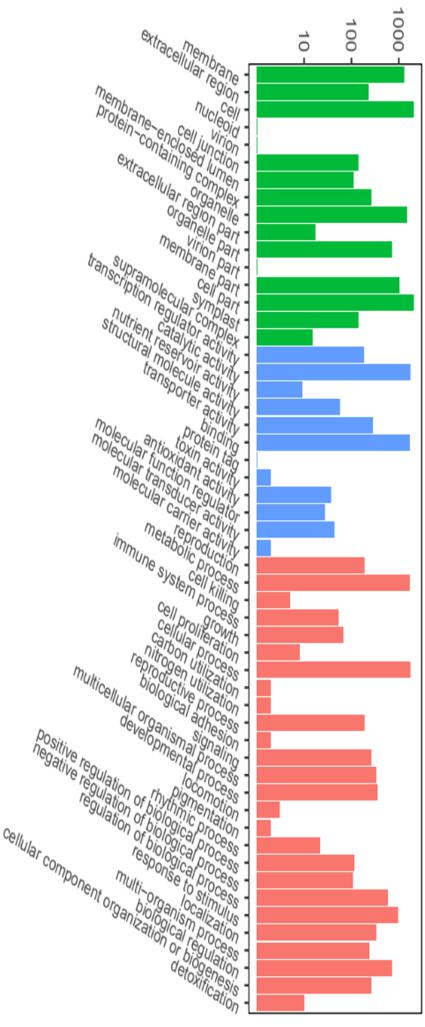

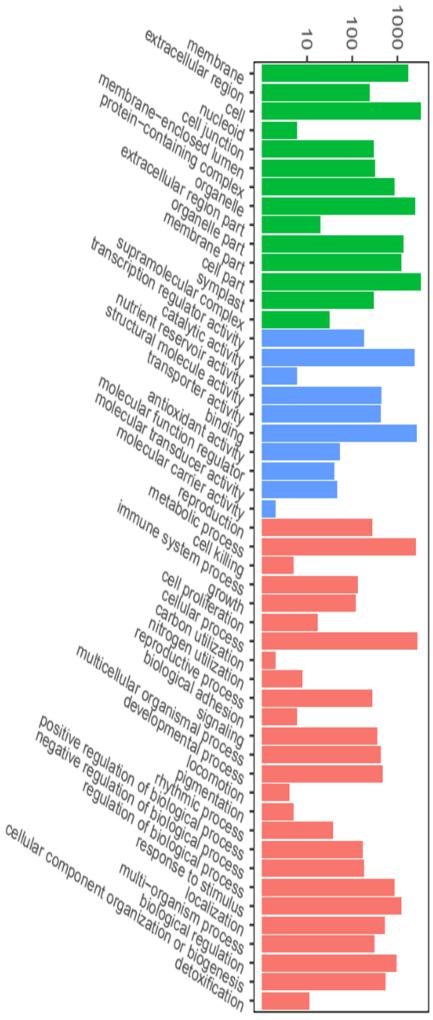

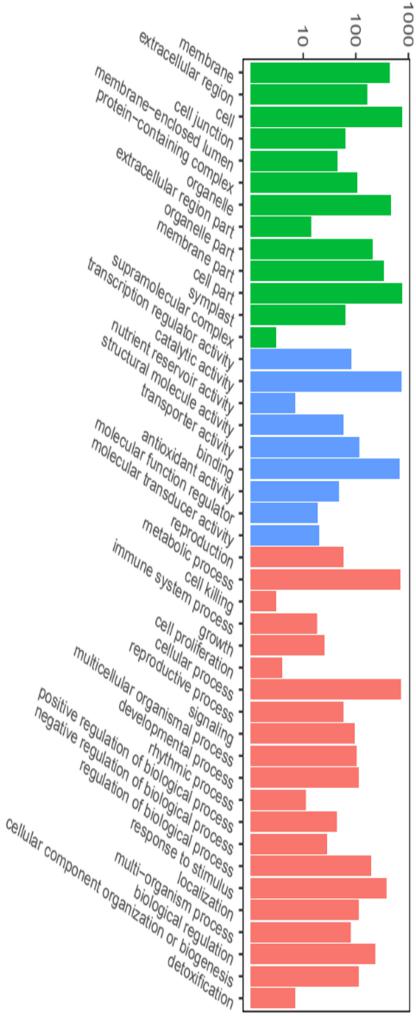


cellular components

molecular functions

biological processes

**Supplementary Figure 3.** GO analysis of upregulated DEGs in **(A)** W0 vs. WJ , **(B)** R0 vs. RJ , and **(C)** WJ vs. RJ.

W0 vs. WJ

R0 vs. RJ


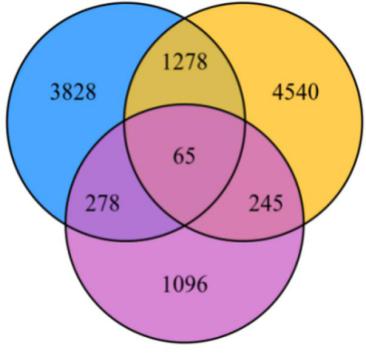


WJ vs. RJ

**Supplementary Figure 4.** Venn diagram of DEGs. The overlapping region of the circles indicates common DEGs among the three combinations.

A

Statistics of KEGG Enrichment


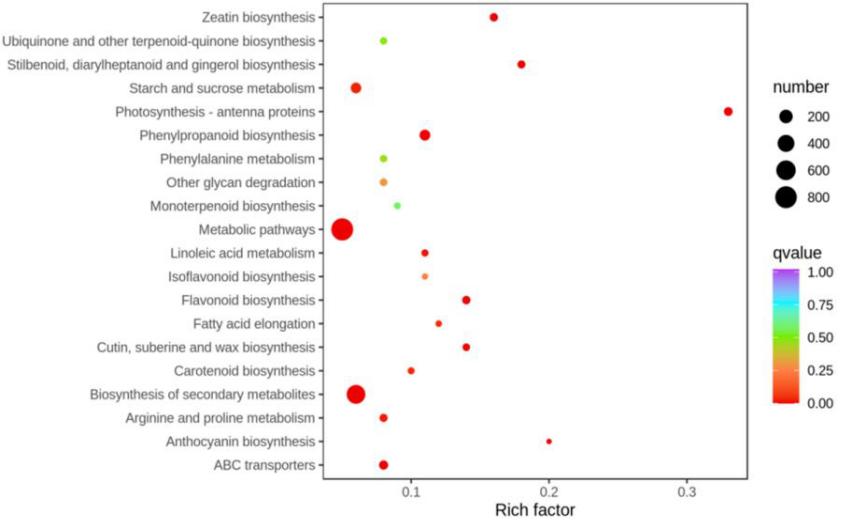


B


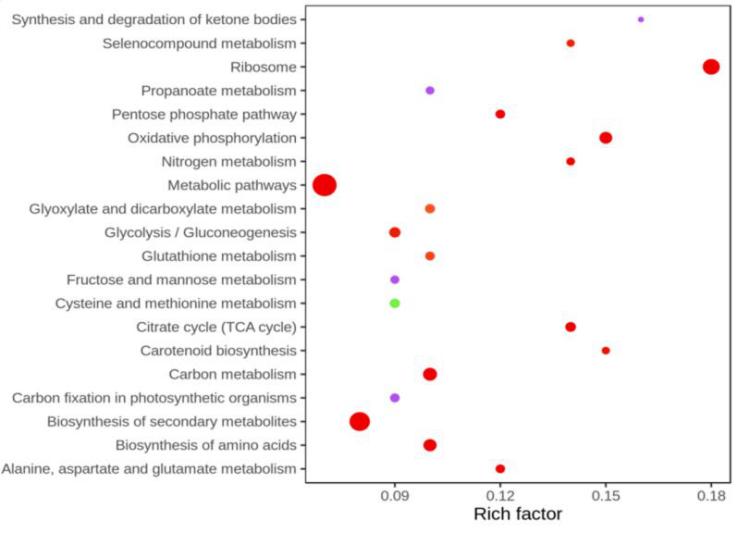


C


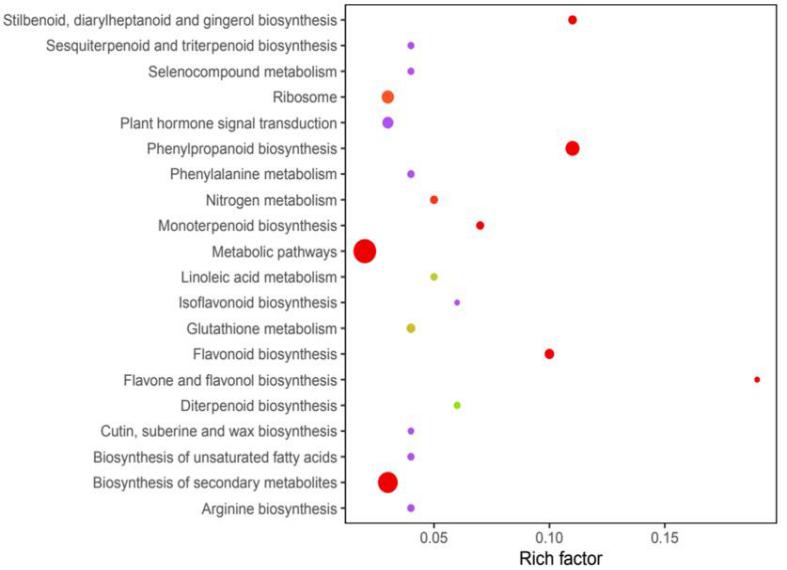


**Supplementary Figure 5.** KEGG pathway enrichment analysis of continuous DEGs in **(A)** W0 vs WJ、**(B)** R0 vs RJ and **(C)** WJ vs RJ**.** The enrichment factor indicates the ratio of DEGs enriched in this pathway to the total number of annotated unigenes. The size and color of each point represents the number of genes enriched in a particular pathway and the q-values, respectively. A larger enrichment factor value and lower q-values shows a greater degree of enrichment. We selected the 20 most significantly enriched pathway for display in this figure.

**Supplementary Table 1 Output quality in data**

| Sample | Raw Reads | Clean reads | Clean bases | Error (%) | Q20 (%) | Q30 (%) | GC (%) |
| --- | --- | --- | --- | --- | --- | --- | --- |
| W0-1 | 54439456 | 56583406 | 7.21 | 0.04 | 95.36 | 89.61 | 42.35 |
| W0-2 | 61428398 | 62933114 | 8.13 | 0.04 | 95.55 | 89.87 | 42.53 |
| W0-3 | 61770522 | 53182862 | 8.43 | 0.04 | 95.28 | 89.45 | 42.32 |
| R0-1 | 53881368 | 47321334 | 7.1 | 0.03 | 95.75 | 90.44 | 42.79 |
| R0-2 | 79116126 | 71166892 | 10.68 | 0.04 | 95.53 | 89.76 | 42.48 |
| R0-3 | 79123908 | 69912478 | 10.49 | 0.04 | 95.49 | 89.86 | 42.69 |
| WJ-1 | 70581018 | 61505336 | 9.23 | 0.03 | 95.74 | 90.4 | 42.91 |
| WJ-2 | 62773642 | 53886718 | 8.08 | 0.03 | 95.89 | 90.6 | 42.39 |
| WJ-3 | 63611882 | 57595304 | 8.64 | 0.04 | 95.46 | 89.64 | 42.50 |
| RJ-1 | 52069694 | 56184430 | 8.43 | 0.03 | 95.74 | 90.27 | 42.32 |
| RJ-2 | 57502978 | 46555074 | 6.98 | 0.04 | 95.89 | 89.29 | 41.76 |

**Supplementary Table 2 The data statistics of gene annotation to each protein databases**

| Database | Number of genes | Percentage（%） |
| --- | --- | --- |
| KEGG | 67279 | 24.67 |
| NR | 96551 | 35.41 |
| SwissProt | 64421 | 23.63 |
| Trembl | 94966 | 34.83 |
| KOG | 53463 | 19.61 |
| GO | 81136 | 29.76 |
| Pfam | 60558 | 22.21 |
| Annotated in at least one Database | 98031 | 35.95 |
| Total Unigenes | 272669 | 100.00 |

**Supplementary Table 3 The expression levels of 7 DEGs**

| Gene-id | W | WJ | Trend | R | RJ | Trend | WJ | RJ | Trend |
| --- | --- | --- | --- | --- | --- | --- | --- | --- | --- |
| Cluster-45258.46315（*NPR1*） | 0 | 3.488 | up | 0 | 1.463 | up | 3.488 | 1.463 | down |
| Cluster-45258.147113（*PAL1*） | 0 | 7.675 | up | 0 | 3.123 | up | 7.675 | 3.123 | down |
| Cluster-45258.100151（*PAL2*） | 0 | 6.081 | up | 0 | 1.797 | up | 6.081 | 1.797 | down |
| Cluster-45258.111810（*TGA1*） | 0 | 6.159 | up | 0 | 4.845 | up | 6.159 | 4.845 | down |
| Cluster-45258.107755（*TGA3*） | 0 | 2.228 | up | 0 | 2.140 | up | 2.228 | 2.140 | down |
| Cluster-45258.62530（*PR1*） | 0 | 13.588 | up | 0 | 7.043 | up | 13.588 | 7.043 | down |
| Cluster-45258.61119（*PR5*） | 0 | 6.865 | up | 0 | 1.828 | up | 6.865 | 1.828 | down |

**Supplementary Table S4 Primers used in this study**

| Primer function | Primer name | Primer sequence (5′-3′) |
| --- | --- | --- |
| Cloned primers | CmNPR1-F  CmNPR1-R | ATGGTGGCTGCATCACAT |
|  |  | TCACGAGTACTTCCGAGATCTGTTCTT |
| qRT-PCR primers | qRT-Actin-F  qRT-Actin-F | TCCGTTGCCCTGAGGTTCT |
|  |  | GATTTCCTTGCTCATCCTGTCA |
|  | qRT-NPR-F  qRT-NPR-R | TGTCGAGAAGGATGGAAAGCC |
|  |  | GGAGGCACCCATCATCAACA |
|  | qRT-PAL1-F  qRT-PAL1-R | GCTCGGAACAGGTTACTTGACAGG |
|  |  | CCATTCCAGCCTTGAAGACACTCC |
|  | qRT-PAL2-F  qRT-PAL2-R | ACAGTCAAGAACACCGTAAGCCAAG |
|  |  | ATACTCACGATCAACCACACGAAGC |
|  | qRT-TGA1-F  qRT-TGA1-R | TCCCTCCATCTTTGCTCTTTCGTTTC |
|  |  | TGATTGACTCCACCTTTGAAGACTGC |
|  | qRT-TGA3-F  qRT-TGA3-R | CTCCAGCACCAACCCATCTTTCG |
|  |  | CTTCGGGCTAACATGAGTCTCGTATG |
|  | qRT-PR1-F  qRT-PR1-R | CCTTAAACCCAATCTCACAATG |
|  |  | TGAGTGCGTTGGTGGAGTT |
|  | qRT-PR5-F  qRT-PR5-R | CCAATGGAGTTTAGCCCCGT |
|  |  | GTCCACAACTACCACGCTCA |
|  | qRT-PR-10-F  qRT-PR-10-R | TCGACTGACATAAGGCACATAACGC |
|  |  | GCTTCCATAACCTCCATCTCCTTCG |
| pTRV2-NPR1 vector construction primers | pTRV2-NPR1-F  pTRV2-NPR1-R | GAATTCAGGCTCTTGTTGAAGATGTGATCC |
|  |  | GGTACCCCTCCTCGGATTTTTCTAAGAGTT |
| hiTAIL-PCR primers | NPR1SP1-F/R  NPR1SP2-F/R  NPR1SP3-F/R | CTCGGATC AGAATCCAAGTCAGTTTC |
|  |  | CGATGGACTGCTGAGTGGCACCTGTACCACAACAACCGCGTCACTA |
|  |  | CAGTACCTAACAAGAACCAAACACCC |
| pCAMBIA1301-NPR1-GUS vector primers | pNPR1.1-F | TACGAATTCGAGCTCGGTACCGAGTGATTCAAATTCCTGGGTAAAG |
|  | pNPR1.1-R | TTACCCTCAGATCTACCATGGCTGCCACGAGAAGGTGATGATGATG |
|  | pNPR1.2-F | TACGAATTCGAGCTCGGTACCCACGCTATCGTATACAAACGCTCCG |
|  | pNPR1.2-R | TTACCCTCAGATCTACCATGGCTGCCACGAGAAGGTGATGATGATG |
|  | pNPR1.3-F | TACGAATTCGAGCTCGGTACCTTACTTGTTGTTTGACAAACGAACAT |
|  | pNPR1.3-R | TTACCCTCAGATCTACCATGGCTGCCACGAGAAGGTGATGATGATG |
| pAbAi-NPR1 vector primers | pNPR1-AbAi-F | CTTGAATTCGAGCTCGGTACCGAGTGATTCAAATTCCTGGGTAAAG |
|  | pNPR1-AbAi-R | TACAGACACATGCCTCGAGACTAATAGATGATGATGGTTCAGCCA |
|  | PA1-AbAi-F | CTTGAATTCGAGCTCGGTACCGAAGGTAGATTTGGTGTTGTGTTGA |
|  | PA1-AbAi-R | TACAGACACATGCCTCGAGACTAATAGATGATGATGGTTCAGCCA |
|  | PA2-AbAi-F | CTTGAATTCGAGCTCGGTACCGAGTGATTCAAATTCCTGGGTAAAG |
|  | PA2-AbAi-R | ATACAGAGCACATGCCTCGAGCATTATCATTCAACACAACACCAAAT |
| pGAD-CmWRKY15-1 vector primers | pGAD-CmWRKY15-1-F | GCCATGGAGGCCAGTGAATTCATGGTGGCTGCATCACATGC |
|  | pGAD-CmWRKY15-1-R | CAGCTCGAGCTCGATGGATTCTTAACATACTTTGAATAAAAAATTACTAC |
| BiFc vector primers | P2YC-CmWRKY15-1-F | CATTTACGAACGATAGTTAATTAAATGGTGGCTGCATCACATGC |
|  | P2YC-CmWRKY15-1-R | CACTGCCACCTCCTCCACTAGTACATACTTTGAATAAAAAATTACTAG |
|  | P2YN-CmNPR1-F | CATTTACGAACGATAGTTAATTAAATGGCTGAACCATCATCATCTATTA |
|  | P2YN-CmNPR1-R | CACTGCCACCTCCTCCACTAGTCGAGTACTTCCGAGATCTGTTCTTCA |
